# Poncirin Induces Apoptosis in AGS Human Gastric Cancer Cells through Extrinsic Apoptotic Pathway by up-Regulation of Fas Ligand

**DOI:** 10.3390/ijms160922676

**Published:** 2015-09-18

**Authors:** Venu Venkatarame Gowda Saralamma, Arulkumar Nagappan, Gyeong Eun Hong, Ho Jeong Lee, Silvia Yumnam, Suchismita Raha, Jeong Doo Heo, Sang Joon Lee, Won Sup Lee, Eun Hee Kim, Gon Sup Kim

**Affiliations:** 1 Research Institute of Life Science and College of Veterinary Medicine (BK21 Plus Project), Gyeongsang National University, Gazwa, Jinju 660-701, Korea; E-Mails: gowdavenu27@gmail.com (V.V.G.S.); hge@gnu.ac.kr (G.E.H.); rock05072000@naver.com (H.J.L.); silviayumnam@gmail.com (S.Y.); suchibiodash@gmail.com (S.R.); 2 Department of Internal Medicine, Institute of Health Sciences, Gyeongsang National University School of Medicine, Jinju 660-702, Korea; E-Mails: arulbiotechtnau@gmail.com (A.N.); lwshmo@gnu.ac.kr (W.S.L.); 3 Gyeongnam Department of Environment Toxicology and Chemistry, Toxicity Screening Research Center, Korea Institute of Toxicology, Jinju 666-844, Korea; E-Mails: jdher@kitox.re.kr (J.D.H.); sjlee@kitox.re.kr (S.J.L.); 4 Department of Nursing Science, International University of Korea, Jinju 660-759, Korea; E-Mail: iuknurse@nate.com

**Keywords:** Poncirin, gastric adenocarcinoma, AGS cells, FasL, caspases, apoptosis

## Abstract

Poncirin, a natural bitter flavanone glycoside abundantly present in many species of citrus fruits, has various biological benefits such as anti-oxidant, anti-microbial, anti-inflammatory and anti-cancer activities. The anti-cancer mechanism of Poncirin remains elusive to date. In this study, we investigated the anti-cancer effects of Poncirin in AGS human gastric cancer cells (gastric adenocarcinoma). The results revealed that Poncirin could inhibit the proliferation of AGS cells in a dose-dependent manner. It was observed Poncirin induced accumulation of sub-G1 DNA content, apoptotic cell population, apoptotic bodies, chromatin condensation, and DNA fragmentation in a dose-dependent manner in AGS cells. The expression of Fas Ligand (FasL) protein was up-regulated dose dependently in Poncirin-treated AGS cells Moreover, Poncirin in AGS cells induced activation of Caspase-8 and -3, and subsequent cleavage of poly(ADP-ribose) polymerase (PARP). Inhibitor studies’ results confirm that the induction of caspase-dependent apoptotic cell death in Poncirin-treated AGS cells was led by the Fas death receptor. Interestingly, Poncirin did not show any effect on mitochondrial membrane potential (ΔΨm), pro-apoptotic proteins (Bax and Bak) and anti-apoptotic protein (Bcl-xL) in AGS-treated cells followed by no activation in the mitochondrial apoptotic protein caspase-9. This result suggests that the mitochondrial-mediated pathway is not involved in Poncirin-induced cell death in gastric cancer. These findings suggest that Poncirin has a potential anti-cancer effect via extrinsic pathway-mediated apoptosis, possibly making it a strong therapeutic agent for human gastric cancer.

## 1. Introduction

Globally, gastric cancer is the second leading cause of cancer-related deaths; the incidence rates for gastric cancer are high in Asian countries such as Korea, China and Japan [1]. Even though survival rates of gastric cancer patients are increasing gradually with advanced techniques, most of the patients die at a late severe stage [2,3]. In addition, gastric cases in the elderly population are increasing in number as they cannot tolerate conventional chemotherapy [4]. Currently, the available treatments for gastric cancer are inadequate. With the recent advanced techniques, the overall give-year survival rate of gastric cancer patients ranges from 10%–30%. However, patients of gastric cancer in severe stages are untreatable [1,2]. Hence, there is urgency to identify novel therapeutic agents that can reduce the mortality of cancer patients with lower side effects.

**Figure 1:**
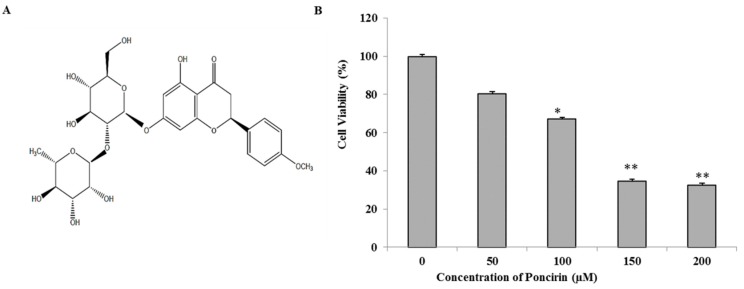
Growth inhibition by Poncirin in AGS human gastric cancer cells (**A**) Chemical structure of Poncirin; (**B**) AGS cells were incubated at indicated concentrations of Poncirin for 24 h. The cell viability was analyzed by 3-(4,5-dimethylthiazol-2-yl)-2,5-diphenyltetrazolium bromide (MTT) assay. The data are shown as means ± standard deviation (SD) of three independent experiments (* *p* < 0.05 and ** *p* < 0.01 *vs.* control).

Natural herbs have gained attraction in the present era by controlling cancer as an alternative therapeutic drug. It has been reported that a high intake of fruit and vegetables, which are rich in flavonoids, is associated with low incidence of cancer [5,6,7]. Therefore, a new natural source of anticancer compounds with relatively fewer side effects would be a valuable tool in cancer therapy. Flavonoids are natural polyphenolic compounds widely occurring in fruits and vegetables. In the recent years, the adoption of flavonoids as anti-cancer compounds has received considerable attention [8]. Citrus fruits contain abundant flavonoids such as Hesperidin, Naringin, Nobiletin, and Poncirin, which exhibit anticancer effect in different cancer cells [9,10]. Poncirin, the 7-*O*-neohesperidoside of isosakuranetin (Figure 1A), is a flavanone glycoside with bitter taste possessing various biological benefits such as antioxidant, anti-microbial, anti-inflammatory, and anti-cancer activities [11,12,13]. However, limited reports are available regarding anti-cancer effects of Poncirin and its molecular mechanisms.

Apoptosis, type 1 programmed cell death, plays a fundamental role in the normal development and differentiation of multicellular organisms, and is a one of the mechanisms by which chemotherapeutic agents induce an anticancer effect and eliminate the affected cells [14,15]. Apoptosis can be initiated by either the death receptor (extrinsic) or mitochondria-dependent (intrinsic) pathways. The mitochondrial pathway is mainly controlled by Bcl-2 family proteins and triggered by the release of cytochrome c due to the loss of mitochondrial transmembrane potential [16]. The extrinsic apoptotic signaling pathways are stimulated by the activation of death receptor (DRs) and mediated by FasL (Fas ligand) and Fas/CD95 receptor protein interaction upon sequential activation of caspase-8 and -3 and poly(ADP-ribose) polymerase (PARP) [17,18,19,20]. Cleavage of cellular substrates degrades the chromosomes into fragments during apoptosis. Ample evidence suggests that apoptosis induced by molecules plays crucial roles in the anticancer properties of many anti-cancer agents by eliminating damaged cells or inhibiting abnormal cell development.

Based on the above evidence, an investigation has been undertaken to understand the mechanism of anti-cancer effect of Poncirin on AGS human gastric cancer cells focusing on both intrinsic and extrinsic apoptotic pathways. Our findings suggest that Poncirin induces apoptosis through extrinsic apoptotic signaling pathway by up-regulation of FasL. To the best of our knowledge, this study is the first report on the anti-cancer effects of Poncirin monomer and its molecular mechanisms against human gastric cancer AGS cells.

## 2. Results

### 2.1. Poncirin Inhibits Proliferation of AGS Human Gastric Cancer Cells

To determine appropriate inhibitory concentrations of Poncirin on AGS cells, cells were treated with various concentrations (50, 100, 150 and 200 µM) of Poncirin for 24 h and the cell viability was measured by a 3-(4,5-dimethylthiazol-2-yl)-2,5-diphenyltetrazolium bromide (MTT) assay. The growth of AGS cells was inhibited by Poncirin in a dose-dependent manner (Figure 1B), and an IC_50_ value of approximately 130 µM at 24 h was observed. The viability of AGS cells decreased gradually viz., 80.46%, 67.13%, 34.54% and 32.58% at 50, 100, 150 and 200 µM doses, respectively. Poncirin showed the cell growth inhibitory effect only in AGS human gastric cancer cells but did not demonstrate any cytotoxicity in normal cells [11]. Therefore, the inhibitory effect of Poncirin is cancer specific. Hence, a lower concentration (50 µM) and a higher concentration (150 µM) dose of Poncirin have been used in the subsequent experiments.

### 2.2. Poncirin Induced Sub-G1 Accumulation and Apoptosis in AGS Cells

**Figure 2:**
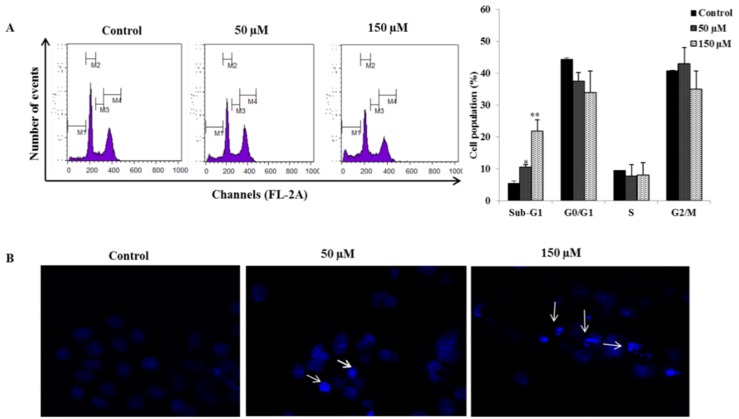
Induction of apoptosis by Poncirin in AGS cells. AGS cells were incubated at indicated concentrations of Poncirin for 24 h. (**A**) Cell cycle analysis and sub-G1 DNA content determination by flow cytometry (M1-Sub-G1, M2-G0/G1, M3-S, M4-G2M phase of cell) (**B**) DAPI staining showing nuclear condensation and fragmentation. White arrows showing bright blue regions indicate nuclear condensation in AGS cells. The data are shown as means ± SD of three independent experiments. (* *p* < 0.05 and ** *p* < 0.01 *vs.* control).

**Figure 3:**
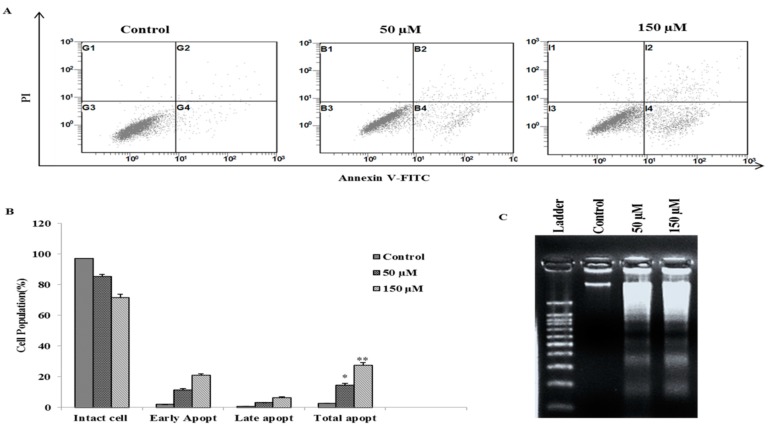
Poncirin induces dose dependent apoptosis in AGS cells. AGS cells were incubated at indicated concentrations of Poncirin for 24 h. (**A**,**B**) Annexin V-Propidium Iodide staining followed by apoptosis detection by flow cytometry; and (**C**) DNA fragmentation test of untreated and Poncirin-treated AGS cells indicates internucleosomal cleavage associated with apoptosis. The data are shown as means ± SD of three independent experiments (* *p* < 0.05 and ** *p* < 0.01 *vs.* control).

Flow cytometry analysis was performed to determine the cell cycle distribution and the population of cell death in Poncirin treated AGS cells. The results show that the sub-G1 DNA content (apoptotic fraction) was significantly increased to 10.62% and 21.87% at 50 and 150 µM of Poncirin, respectively (Figure 2A). The effect of Poncirin on the induction of apoptosis in AGS cells was assessed by Annexin V-FITC/PI double-labeled flow cytometry. Poncirin treatment significantly increased the total apoptosis from 2.7%, 14.4% and 27.3% at 0, 50 and 150 µM concentrations, respectively (Figure 3A,B). Moreover, DNA fragmentation analysis revealed a typical ladder pattern of fragmented DNA, which indicates internucleosomal cleavage, associated with apoptosis (Figure 3C). The DAPI (4′,6-Diamidino-2-phenylindole) staining also revealed a dose-dependent increase in the number of apoptotic cells of Poncirin-treated AGS cells (Figure 2B). These results clearly suggest that Poncirin could induce apoptotic cell death in AGS cells.

### 2.3. Poncirin Induced Apoptosis via FasL Dependent Extrinsic Apoptotic Pathway in AGS Gastric Cancer Cells

**Figure 4:**
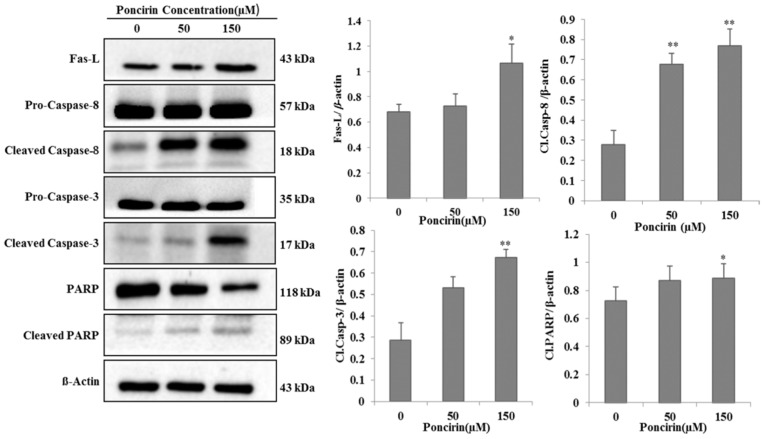
Activation of caspases, subsequent cleavage of PARP and up-regulation of Fas ligand in the Poncirin treated AGS cells. AGS cells were incubated at indicated concentrations of Poncirin for 24 h. Western blot analysis was conducted to determine the effects of Poncirin on the caspase activation, and PARP cleavage and Fas ligand up-regulation was measured by protein expression. β-Actin served as a loading control (* *p* < 0.05 and ** *p* < 0.01 *vs.* control group).

FasL is an apoptotic ligand and it triggers the extrinsic apoptotic pathway. To determine involvement of extrinsic apoptotic pathway in Poncirin-induced apoptosis, the expression of FasL was measured by immune blotting. Western blot analysis revealed that Poncirin up regulates FasL in a dose-dependent manner (Figure 4). Caspase-8 and -3 are downstream targets of FasL, which were significantly activated by Poncirin in a dose-dependent manner (Figure 4). Poncirin also induced the cleavage of PARP, which is a substrate of activated caspase-3.

**Figure 5:**
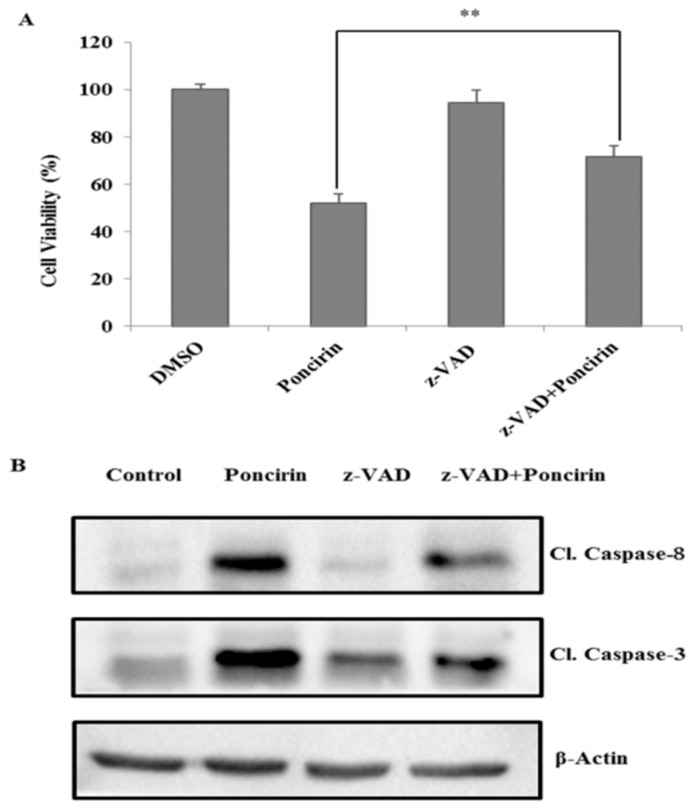

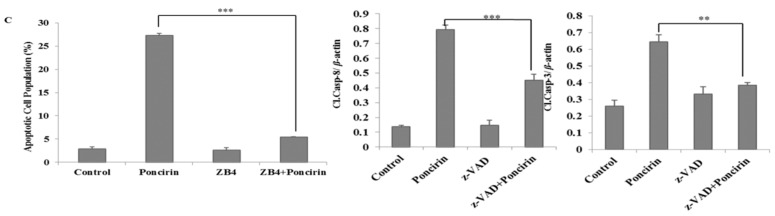
Poncirin activates extrinsic apoptosis pathway in AGS cells. (**A**) Cell viability of AGS cells with or without z-VAD (z-VAD-fmk), AGS cells were treated with 10 µM of z-VAD-fmk for 1 h before Poncirin treatment. The cell viability was determined by MTT assay; and (**B**) caspase-8 and caspase-3 protein expression was determined by Western blotting. β-Actin served as a loading control; and (**C**) AGS cells were pretreated with 500 ng/mL of ZB4 monoclonal antibody for 1 h and followed by Poncirin treatment for 24 h. Extent of apoptosis was measured by Annexin V-Propidium Iodide Apoptosis Detection by flow cytometry. (*** *p* < 0.01, ** *p* < 0.05 *vs.* Poncirin-treated group).

Further, to confirm Poncirin induced apoptosis is a caspase and Fas death receptor-dependent pathway, an inhibitory assay was undertaken. Poncirin induced cell cytotoxicity in AGS gastric cancer cells was partially recovered by pretreatment with pan caspase inhibiter Z-VAD-fmk as compared with Poncirin treated cells (Figure 5A). Moreover, the cleavages of caspase-8 and caspase-3 were significantly inhibited with z-VAD-fmk pretreatment, while the Poncirin alone still led to the cleavage of caspase-8 and caspase-3, suggesting that poncirin induced cell death is caspase dependent (Figure 5B). Further, to confirm the effect of Poncirin on Fas mediated cell death, AGS cells were pretreated with 500 ng/mL of ZB4 clone as Fas inhibitor. It was observed that there was partial recovery of Poncirin induced cell cytotoxicity in AGS gastric cancer cells (MTT assay results not shown) and there was a significant decrease in the total apoptotic population compared with the Poncirin treated group (Figure 5C). Collectively, these results suggest that the apoptotic cell death induced by Poncirin in AGS cells occurs via the Fas death receptor mediated and caspase dependent extrinsic apoptosis pathway.

### 2.4. Poncirin Induced Apoptotic Cell Death Is Independent of Intrinsic Mitochondrial Apoptotic Pathway in AGS Cells

**Figure 6:**
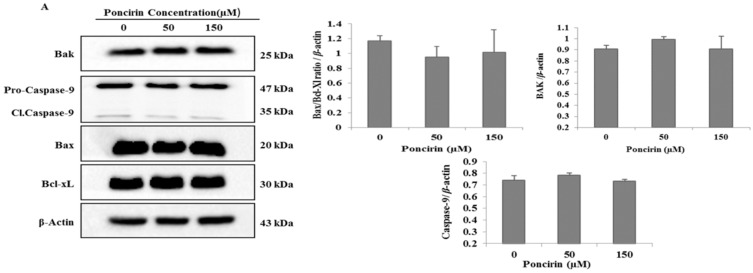

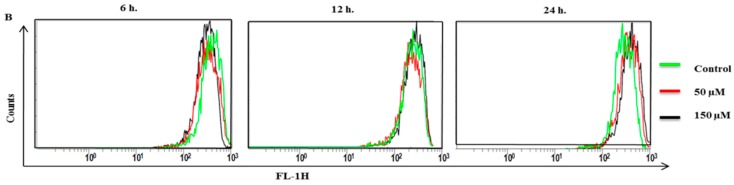
Effects of Poncirin on MMP (ΔΨm), and mitochondrial apoptosis regulatory proteins in AGS cells (**A**).Western blot analysis for the effects of Poncirin on Bak, caspase-9, Bax and Bcl-xL. The results are from one representative of three independent experiments that showed a similar pattern; and (**B**) AGS cells were incubated at indicated concentrations of Poncirin for 6, 12 and 24 h. Cells were stained with DiOC6 (3,3′-dihexyloxacarbocyanine iodide) dye and analysis were done by flow cytometry.

To determine whether Poncirin induced intrinsic mitochondrial apoptotic pathway in AGS cells, immunoblotting of mitochondrial related apoptotic proteins was performed. Figure 6A presents the protein expression level of Bcl-xL and Bax (Bax/Bcl-xL) relative ratio, which was unchanged compared with control. Bak, a pro-apoptotic member of the Bcl-2 family, upon stimulation with BID induces conformational changes in Bak to form oligomer channels in the mitochondrial membrane for cytochrome-c release. Western blot analysis revealed that the protein level of Bak was unchanged in Poncirin treated cells. In addition, the mitochondrial apoptotic pathway related protein caspase-9 was not activated by Poncirin treatment in AGS cells (Figure 6A).

To Further confirm the Western blot data, loss of mitochondrial membrane potential (ΔΨm) was measured using DiOC6 (3,3′-dihexyloxacarbocyanine iodide) dye. Cells treated with various concentrations of Poncirin (0, 50 and 150 µM) for 6, 12 and 24 h and change in fluorescence intensity was measured with FACs. It was observed that Poncirin treated AGS cells did not alter the mitochondrial membrane potential (ΔΨm) activity in both a time and dose dependent manner (Figure 6A). These results suggest that mitochondrial intrinsic apoptotic pathway was not involved in Poncirin-induced apoptosis in AGS cells.

## 3. Discussion

Over thousands of years, citrus spp. have been used most commonly as a traditional medicine in Asian countries including Korea. Numerous flavonoids compounds from citrus fruits like Naringin, Nobiletin and Hesperidin exhibit anti-cancer effect by cell cycle arrest, apoptosis, autophagy and paraptosis pathways in different human cancer cell lines [21,22,23]. The present study was designed to elucidate the mechanisms by which Poncirin induces apoptosis in AGS human gastric cancer cells. This study mainly focuses on the two major apoptotic pathways (intrinsic pathway and extrinsic pathway). Firstly, Poncirin strongly inhibited cell growth and induced cell death of AGS cells in a dose-dependent manner. Secondly, the inhibitory effect of Poncirin was found to involve caspase activation. Poncirin-induced cell death occurred through an extrinsic pathway by up-regulation of FasL, which is independent of the mitochondrial-mediated apoptotic pathway.

The regulation of apoptosis is an important process during development and aging and to maintain cell homeostasis [24]. Apoptosis involves a series of biochemical events leading to a characteristic cell morphology and cell death. Two main molecular signaling pathways have characterized cell apoptosis (intrinsic pathway and extrinsic pathway) [18,24,25]. The cell viability assay revealed that Poncirin significantly inhibited proliferation of AGS cells compared with untreated control cells at 150 µM concentration. It has also been observed that Poncirin does not show any cytotoxicity in normal cells [11]. Thus, in this study, Poncirin inhibiting AGS cell proliferation confirmed its cancer specific inhibition. Recent studies demonstrated that alterations in cell cycle often lead to apoptosis in many cell lines [26,27]. In our present study, AGS cells treated with Poncirin significantly accumulated in the sub-G1 phase (apoptotic cell population), whereas the S phase and G2/M populations were decreased, leading to apoptosis. Inconsistent with the above results, Annexin V-propidium iodide apoptosis detection showed an increase in total apoptotic cell population in a dose dependent manner. A typical ladder pattern of DNA fragmentation and increase in the number of apoptotic bodies also were observed in Poncirin-treated AGS cells. Similarly, several published reports on induction of apoptotic cell death in various cancer cell lines support our obtained results [28,29,30]. Thus, these findings suggest that Poncirin induced cell death in AGS cells occurred due to apoptosis.

Further, the demonstrated results revealed that Poncirin induced apoptosis through an extrinsic pathway by up-regulation of FasL, via sequential activation of caspase-8 and -3, and PARP cleavage, a substrate protein of activated caspase-3 [31]. In this study, Poncirin in AGS cells significantly up-regulates the expression of FasL, which subsequently triggers the activation of apoptotic factors, including caspase-8 and -3 leading to cleavage of PARP. To determine whether the apoptotic process was occurring in a caspase-dependent manner, we pre-treated AGS cells with z-VAD-fmk, which is a known pan-caspase inhibitor [32]. Observed data revealed that the growth inhibition induced by Poncirin was partially abrogated by z-VAD-fmk pretreatment, and it inhibited the increased protein expression of cleaved caspase 8 and 3. Our results determined that Poncirin induced apoptosis in AGS cells relies on a Fas dependent extrinsic pathway, which was elucidated by neutralizing the Fas pathway upon pretreatment of cells with ZB4 clone monoclonal antibody (Fas I), a known Fas neutralizer [33]. A significant decrease in total number of apoptotic cells in ZB4 pretreated AGS cells has been observed compared to the Poncirin alone treated group showing Fas I as rescued apoptosis by Poncirin. Collectively, these results suggest that apoptotic cell death induced by Poncirin in AGS cells was mediated by Fas death receptor followed by the caspase-dependent extrinsic apoptosis pathway.

In contrast, the mitochondrial-dependent apoptosis pathway was not activated in Poncirin-treated AGS cells. Overexpression of Bcl-xL, an anti-apoptotic protein, prevents cells from initiating apoptosis, while an increase of Bax, a pro-apoptotic protein, induces the intrinsic apoptosis pathway [34]. In the present study, we found that the levels of Bax and Bcl-xL were unchanged with Poncirin treatment. The ratio of Bcl-xL/Bax was also unchanged. Moreover, of the intrinsic apoptotic caspases, caspase-9 was not activated in Poncirin-treated AGS cells. In several previous studies, it was found that some phytochemicals induce apoptosis by alteration of MMP (ΔΨm) in various cancer cells [35,36,37]. Interestingly, Poncirin did not show any effect on MMP (ΔΨm) in neither a time- nor dose-dependent manner. These results conclude that the mitochondrial-mediated pathway is not involved in Poncirin-induced apoptosis in AGS gastric cancer cells. Altogether, our findings clearly suggest that Poncirin-induced apoptotic cell death occurs through extrinsic pathways in AGS cells.

## 4. Material and Methods

### 4.1. Chemicals and Reagents

AGS human gastric cancer cells obtained from the Korea Cell Line Bank (Seoul, Korea) were cultured in RPMI 1640 medium supplemented with 10% (*v*/*v*) fetal bovine serum (FBS) from GIBCO (BRL Life Technologies, Grand Island, NY, USA), 100 U/mL penicillin, and 100 μg/mL streptomycin at 37 °C in a humidified atmosphere of 95% air and 5% CO_2_. Poncirin compound purchased from Sigma–Aldrich (St. Louis, MO, USA). 3-(4,5-Dimethylthiazol-2-yl)-2,5-diphenyltetrazolium bromide (MTT) was obtained from Sigma–Aldrich (St. Louis, MO, USA). Materials and chemicals used for electrophoresis were obtained from Bio-Rad (Hercules, CA, USA). Primary antibodies to Bcl-xL, Bax, Bak, caspase (3,-8,-9), cleaved-caspase (3,-8,-9), poly ADP ribose polymerase (PARP), cleaved-PARP, FasL and β-actin were purchased from Cell Signaling Technology (Danvers, MA, USA). Horseradish peroxidase-(HRP-) coupled goat anti-mouse IgG and anti-rabbit IgG were purchased from Enzo Life Sciences. Propidium iodide (PI) purchased from Sigma–Aldrich (St. Louis, MO, USA). DAPI (4′,6-Diamidino-2-phenylindole) purchased from Vector Laboratories Inc. (Burlingame, CA, USA). z-VAD-fmk was purchased from Enzo Life Sciences, Inc. (Farmingdale, NY, USA). Anti-Fas clone ZB4 was purchased from Millipore (Temecula, CA, USA). 6× Agarose Gel Loading Buffer were purchased from Bioneer (Daejeon, Korea), DNA marker was purchased from iNtRON Biotechnology (Kyungki-Do, Korea).

### 4.2. Cell Viability Assay

Overnight grown AGS cells were stabilized for 6 h in serum free RPMI-1640 and were treated with different concentrations with or without Poncirin. Cell viability assay was performed using MTT assay, for which, AGS cells were seeded at a density of 1 × 10^5^cells/well in 12 well plates and then treated with 50, 100, 150 and 200 µM of Poncirin or vehicle (DMSO) alone for 24 h. After incubation, 100 mL of 0.5% (*w*/*v*) MTT dissolved in 1× PBS was added to each well and incubated for 3 h at 37 °C in the dark. The formazan contained in the cell was solubilized by 500 µL of DMSO and the absorbance was measured at 540 nm with an enzyme-linked immunosorbent assay (ELISA) plate reader (BioTek Instruments Co., Seoul, Korea).

### 4.3. Flow Cytometry Analysis for Cell Cycle Analysis

AGS cells were collected after incubation with Poncirin at the indicated concentrations for 24 h, After incubation, cells were washed with ice-cold PBS, trypsinized and collected in a 15 mL conical tube and pelleted by centrifugation (1000× *g*) for 5 min. The cells were than fixed in 70% (*v*/*v*) ethanol for 1 h at 4 °C. The cells were washed with PBS and stained with propidium iodide (50 μg/mL) including RNase A (0.1 mg/mL) in PBS (pH 7.4) for 30 min in the dark. Flow cytometry analyses were performed with FACS Calibur flow cytometer (BD Biosciences, Franklin Lakes, NJ, USA). In each sample, 10,000 cells were sorted. The data were analyzed using Cell Quest software (Becton Dickinson, NJ, USA).

### 4.4. Annexin V-Propidium Iodide Apoptosis Detection

Apoptotic cells were detected by using a FITC Annexin-V apoptosis detection kit 1 (BD Pharmingen, San Diego, CA, USA). Briefly, after treatment with various concentrations of Poncirin (0, 50 and 150 μM), for 24 h, the cells were collected and washed with PBS, re-suspended in binding buffer, and stained with Annexin V-FITC and PI for 15 min at room temperature in the dark, prior to the addition of binding buffer. Flow cytometry analyses were performed with Cytomics FC 500 (Beckman Coulter, Brea, CA, USA). In each sample, 10,000 cells were sorted. The data were analyzed by using *CXP* Software (Beckman Coulter, Inc., Fullerton, CA, USA).

### 4.5. Nuclear Staining with DAPI

After 24 h incubation with Poncirin at the indicated concentrations, cells were washed with phosphate-buffered saline (PBS). Cells were fixed with 37% formaldehyde and 95% ethanol (1:4) for 10 min at room temperature followed by washing the cells with PBS and stained with 2.5 μg/mL of DAPI solution for 10 min at room temperature (RT). The stained cells were than examined through a fluorescence microscope from Leica Microsystems Ltd. (Seoul, Korea).

### 4.6. DNA Fragmentation Assay

After 24 h incubation with Poncirin at the indicated concentrations, the cells were harvested and lysed in a lysis buffer (1% NP-40 in 20 mM EDTA, 50 mM Tris-HCl, and pH 7.5) for 30 s at RT (room temperature). Centrifuged the lysed cells at 3000 rpm for 5 min and supernatant was collected. Pooled supernatant was incubated for 2 h at 56 °C after adding 10 µL of 10% SDS solution (final concentration: 1% SDS) and 10 µL of 50 mg/mL RNaseA (final concentration 5 µg/µL). Then, 10 µL of Proteinase K (final concentration 2.5 µg/µL) was added and incubated for 2 h at 37 °C, followed by adding 1/2 volume (65 µL) of 10 M NaCl and 2.5 volume (500 µL) of ice-cold ethanol and mixed thoroughly. The mixture was incubated for 1 h in −80 °C (ethanol precipitation). It was then centrifuged for 20 min at 12,000 rpm followed by washing the white pellet with 200 µL of 80% ice-cold ethanol and air-dried for 10 min at room temperature. The pellet was dissolved with 50 µL of TE buffer and the concentration of DNA was determined using Nano drop. Electrophoretic analysis was performed on 1.5% agarose gels containing 0.1 μg/mL EtBr (ethidium bromide) .Tris acetate EDTA was used as the electrophoresis-running buffer and DNA bands were visualized by UV light and documented by photography.

### 4.7. Inhibitor Assay

To confirm the role of Fas pathway and caspases in Poncirin induced apoptosis, inhibitor studies were carried out. The cells were incubated with ZB4 clone as a Fas inhibitor (Fas I) at 500 ng/mL or broad-spectrum caspase inhibitor z-VAD-fmk at 20 µM concentration 1 h prior to Poncirin treatment. After the incubation with Poncirin for 24 h, the cells were processed for cell viability with MTT assay and Annexin V-Propidium Iodide staining was used to measure the percentage of apoptosis. Western blot analysis was undertaken to measure the activity of caspase-8 and caspase-3.

### 4.8. Measurement of Mitochondrial Membrane Potential (MMP, ΔΨm)

AGS cells were incubated with subsequent doses of Poncirin for different time periods at 37 °C in complete media and were harvested and washed with 1× PBS. Washed cells were stained with DiOC_6_ (3,3′-dihexyloxacarbocyanine iodide) dye, and change in fluorescent intensity was analyzed using a flow cytometer (FACS Calibur BD Biosciences, Franklin Lakes, NJ, USA).

### 4.9. Western Blot Analysis

AGS cells were treated with Poncirin at 0, 50 and 150 μM for 24 h at 37 °C and were lysed in RIPA buffer [1% (*w/v*) NP40, 1% (*w/v*) sodium deoxycholate, 0.1% (*w/v*) SDS, 0z.15 M NaCl, 0.01 M sodium phosphate buffer, pH 7.2, 2 mM EDTA, and 50 mM phosphatase inhibitor cocktail]. The cell extracts were centrifuged for 30 min at 12,000 rpm to remove debris. Bradford assay (Bio-Rad) method was used to quantify proteins. Proteins were separated by 8%–12% SDS-polyacrylamide gel electrophoresis (SDS-PAGE) and transferred to a polyvinyldene fluoride (PVDF) membrane (Immunobilon-P, 0.45 mm; Millipore, Billerica, MA, USA) using the TE 77 Semi-Dry Transfer Unit (GE Healthcare Life Sciences, Buckinghamshire, UK). The membranes were blocked with 5% non-fat skim milk in Tris buffered saline containing 1% Tween 20 (TBS-T, pH 7.4) at room temperature for 1 h, and incubated overnight at 4 °C with a 1:1000 dilution of respected primary antibody. The membranes have washed five times with TBS-T for 10 min each at room temperature, incubated with a 1:2000 dilution of HRP conjugated secondary antibody for 3 h at room temperature. The membranes were then rewashed five times with TBS-T. Blots were developed with ECL detection system (GE Healthcare Life Science). The bands were quantitatively analyzed by using the Image J program (http://rsb.info.nih.gov). The densitometry readings of the bands were normalized according to β-actin expression.

**Figure 7:**
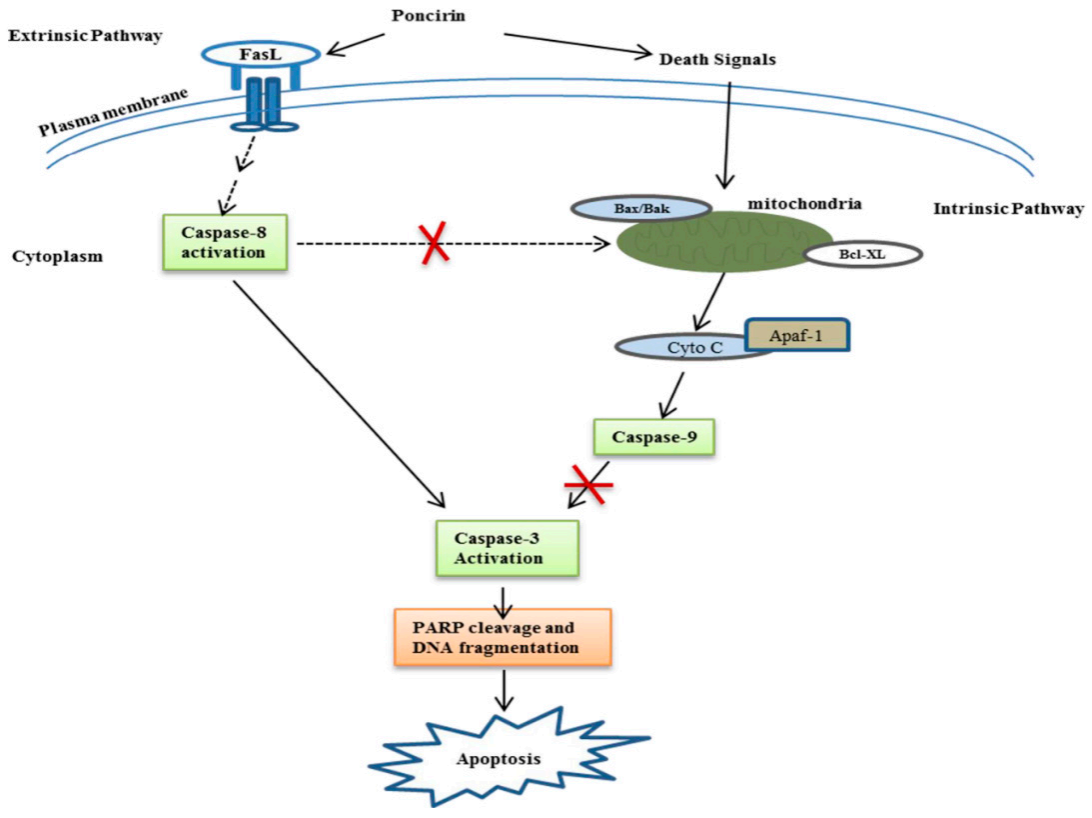
Schematic diagram showing the mechanisms underlying the anti-cancer effects of Poncirin. Poncirin induced apoptosis by up-regulating FasL, and then activated caspase-8 and -3, which subsequently activated cleavage of PARP and DNA fragmentation. Poncirin does not alter MMP (ΔΨm) and mitochondrial regulatory proteins in AGS cells. Taken together, this study suggests that Poncirin induced apoptosis through extrinsic pathways in AGS cells, which is independent of mitochondrial apoptotic pathway. (→ indicates activation, ⊥ indicates inhibition, --- indicates indirect or multiple pathways, **×** indicates pathway blocked).

### 4.10. Statistical Analysis

Each experiment was performed in triplicate and data were expressed as the mean ± standard deviation (SD). Differences between the control and Poncirin treated groups were determined using one-way analysis of variance followed by a Student’s *t*-test with *p* < 0.05 and *p* < 0.01 as the limit of significance. All statistical analyses were performed using SPSS software (SPSS for Windows, v. 10.0; SPSS Inc., Chicago, IL, USA).

## 5. Conclusions

In conclusion, the present study clearly demonstrates that Poncirin induced apoptosis via an extrinsic apoptotic pathway in AGS human gastric cancer cells by up-regulation of FasL, which is independent of mitochondrial-related apoptotic pathways (Figure 7). These findings highlight the therapeutic potential of Poncirin and its molecular mechanism for anti-cancer activity. Thus, it should be evaluated further as a therapeutic agent in human gastric cancer.
